# Parenting stress among parents of outpatients with precocious puberty: a cross-sectional survey

**DOI:** 10.3389/fpsyt.2026.1688507

**Published:** 2026-02-24

**Authors:** Wei Wei, Xiaojie Zhai, Jia Zou, Dan Chen, Yiwen Zhang

**Affiliations:** Department of Nursing, Children’s Hospital of Nanjing Medical University, Nanjing, Jiangsu, China

**Keywords:** care, clinical, nursing, outpatients, parenting stress, precocious puberty

## Abstract

**Background:**

Precocious puberty imposes notable psychological pressure on parents, yet specific patterns of their parenting stress and related influencing factors remain insufficiently explored. This study aimed to evaluate the parenting stress among parents of outpatients with precocious puberty, to provide insights for clinical nursing care.

**Method:**

A cross-sectional study was conducted between January 2024 and June 2025, enrolling parents of outpatients diagnosed with precocious puberty. Data were collected using the Parenting Stress Index to assess parenting stress levels and a self-designed questionnaire for sociodemographic information.

**Results:**

A total of 236 parents were included. The mean total parenting stress score was 114.85 ± 19.02 points, exceeding the high-stress threshold and indicating pronounced strain. The Parental Distress dimension scored highest (41.02 ± 8.40), followed by Difficult Child (38.18 ± 7.99) and Parent-Child Dysfunctional Interaction (36.79 ± 8.22). Parenting stress showed significant positive associations with maternal caregiver status, rural residence, lower parental education, divorced marital status, lower household income, and a greater number of children. Multiple regression analysis revealed that family income, number of children, marital status, residence, and educational level collectively explained 58.7% of stress variance (R²=0.587, P<0.001).

**Conclusion:**

Parents of children with precocious puberty face significantly elevated stress, with notable sociodemographic disparities. These findings suggest that tailored support strategies—potentially including personalized psychological counseling, simplified disease education, enhanced social support connections, and family-centered approaches—may be beneficial to reduce stress, strengthen parental coping skills, and improve overall care for affected children.

## Introduction

Precocious puberty, as an abnormal condition in children’s growth and development, has been attracting increasing attention from all sectors of society (1). Epidemiologically, central precocious puberty (CPP)—the most common form—affects approximately 1–2 per 10,000 girls and 1 per 50,000 boys worldwide, with notably higher prevalence rates (e.g., 1 in 5,000–10,000 girls) reported in urban regions of China (2, 3). Physiologically, precocious puberty accelerates the skeletal maturation of affected children, leading to premature closure of epiphyses, which in turn severely impacts their final adult height (2). The majority of children with precocious puberty have a significantly lower final height compared to the normal population (4). This dual burden—on final height and, often, on the child’s emotional well-being due to early maturation—underscores the need for comprehensive attention to the condition beyond medical management alone.

Although the impact of precocious puberty on the families of affected children is extensive and profound, with parents reporting significant anxiety, guilt, and marital strain. Psychologically, parents often experience intense and complex emotions, including anxiety about their child’s prognosis, guilt related to potential etiological factors, and helplessness in navigating the medical and caregiving journey (5). Such stressors can strain marital relationships, with conflicts emerging over caregiving responsibilities, treatment decisions, or financial pressures (6). Conflicts and quarrels may also arise between parents over their children’s problems, leading to strained family relationships (7). Notably, parenting stress in this context is further shaped by cultural contexts and health system disparities. In China, for instance, pediatric endocrine services are disproportionately concentrated in urban tertiary hospitals, leaving rural and lower-tier regions with limited access to specialized care (8). Such disparities mean parents in resource-scarce areas face greater barriers to information and treatment, exacerbating their stress (9). Additionally, sociodemographic factors (e.g., parental education, household income, marital status) and psychosocial variables (e.g., coping styles, social support) act as key confounders (10); for example, parents with lower literacy may struggle to understand medical guidance, increasing uncertainty and distress (11). Pubertal characteristics of children, such as the timing and severity of physical changes, also mediate parenting stress by influencing care demands and emotional responses (12).

In this study, ‘parenting stress’ specifically refers to the stress perceived within the parent-child relationship system, encompassing distress from the parenting role, dysfunctional parent-child interaction, and perceptions of a difficult child. Assessing parenting stress in this population is crucial as it can directly affect treatment adherence, family dynamics, and the child’s psychosocial adjustment, thereby influencing overall disease management outcomes. However, systematic research on the parenting stress endured by the parents of affected children is relatively scarce. Therefore, this study aims to assess the current status of parenting stress among parents of outpatients with precocious puberty and identify its influencing factors, thereby offering evidence-based insights to inform the optimization of clinical nursing interventions.

## Methods

### Study design

This study adopted a cross-sectional survey design, targeting parents of children diagnosed with precocious puberty in the Endocrinology Outpatient Department of a tertiary grade A children’s hospital. Data were collected via questionnaire surveys. The design aimed to systematically describe the current status of parenting stress among parents of outpatient children with precocious puberty and analyze its influencing factors through a one-time data collection, thereby providing a basis for formulating targeted nursing strategies.

### Ethical consideration

This study was reviewed and approved by the Ethics Committee of the Children’s Hospital of Nanjing Medical University, with the approval number: 202512054-1. Concurrently, written informed consent was obtained from the included parents.

### Study participants

The participants were parents of children with precocious puberty who attended the Endocrinology Outpatient Department of Nanjing Children’s Hospital (a tertiary grade A facility) from January 2024 to June 2025. This recruitment period was selected to capture seasonal stability in clinic attendance and avoid disruptions from major holidays, ensuring consistent access to the target population. The clinic serves approximately 500 precocious puberty patients annually, with 60% of referrals from urban areas and 40% from rural regions—demographics consistent with the final study sample, supporting the representativeness of the participants.

Inclusion criteria: (1) The child met the diagnostic criteria for precocious puberty (development of secondary sexual characteristics before 8 years of age in girls or before 9 years of age in boys); (2) The parents were the primary caregivers and lived with the child; (3) The parents had basic reading and comprehension abilities to complete the questionnaire independently (standardized assistance was provided for those with limited literacy to avoid exclusion due to literacy barriers); (4) The parents provided informed consent and voluntarily participated in the study. Exclusion criteria: (1) The child had other severe physical diseases or mental disorders; (2) The parents had severe mental illnesses or cognitive impairments; (3) The family had experienced major changes in the past 3 months (e.g., death of a family member, major disasters, etc.).

Only one primary caregiver per child was invited to participate. In cases where multiple children from the same family attended the clinic, only the parent of the first-encountered child was recruited to maintain statistical independence of observations.

### Sample size calculation

The sample size calculation in this study was based on the principle of sample size estimation for multivariate analysis, considering the relationship between the number of independent variables and the sample size. According to relevant research experience (13), the sample size in multiple regression analysis should be at least 10 times the number of independent variables. Based on a preliminary literature review, this study was expected to include approximately 11 independent variables. Calculated as 10 times the number of independent variables, the required sample size was 110 cases. Additionally, considering a 20% invalid or dropout rate of questionnaires, the final sample size was determined to be at least 132 parents to ensure the stability and validity of the study results.

### Research instruments

This study collected data using a combination of a self-designed general information questionnaire and standardized scales, as detailed below:

1. General Information Questionnaire: This questionnaire was developed to collect socio-demographic data, including the child’s gender and age, parents’ age, educational level, occupation, and monthly household income, among other relevant variables.
2. Parenting Stress Index-Short Form (PSI-SF): The original PSI-SF was developed by Abidin (1995), and the Chinese version validated by Lu et al. (14) was used. The short form was selected over the full PSI to minimize participant burden, a critical consideration for clinic-based recruitment where parents have limited time; importantly, it retains the 3 core dimensions of the full scale (Parental Distress, Parent-Child Dysfunctional Interaction, Difficult Child) while reducing item count from 120 to 36, ensuring efficient data collection without compromising key measurement domains.

The PSI-SF comprises 36 items (12 per dimension) and is designed for parents of children aged 1 month to 12 years. It uses a 5-point Likert rating system (1 = “strongly disagree,” 5 = “strongly agree”). Scores for each dimension range from 12 to 60 points, and the total score (sum of all three dimensions) ranges from 36 to 180 points. A total score >90 points indicates high parenting stress (needing counseling/support or potential child neglect risk); ≤85 points reflects normal stress; 86–90 points indicate borderline high stress; and ≥99 points signify extremely high stress (15).

To minimize potential biases associated with self-report tools (e.g., social desirability bias, subjective interpretation, under- or over-reporting), the following quality control measures were implemented: The PSI-SF was selected for its established psychometric robustness in Chinese populations (16), with a Cronbach’s α coefficient of 0.91 for the total scale, 0.87 for Parenting Distress, 0.80 for Parent-Child Dysfunctional Interaction, and 0.85 for Difficult Child (16).; test-retest reliability ranges from 0.68 to 0.85 (17), ensuring the scale’s reliability and validity for assessing subjective stress. All researchers received standardized training on neutral probing techniques to avoid suggestive cues when assisting participants, and responses were verified through repetition to ensure accuracy. Anonymous data collection was adopted to reduce social desirability bias, as participants were not required to provide personally identifiable information beyond basic socio-demographic details necessary for analysis.

### Data collection

Questionnaire administration mode: Data were collected via paper-based questionnaires, distributed and retrieved on-site during clinic visits to ensure immediate clarification of doubts and high response completeness. The distribution and collection of questionnaires were conducted by uniformly trained researchers to ensure standardization. Training content included the research background, questionnaire structure and item meanings, standardized expression of instructions, and contingency plans for common problems. All researchers passed an assessment before participating in data collection to minimize information bias from operational differences.

Questionnaires were distributed in the waiting area of the Endocrinology Outpatient Department, during time periods when children attended consultations accompanied by their parents. It is acknowledged that recruitment from a hospital waiting area may potentially underrepresent parents with extreme psychological distress (as no formal distress screening was conducted); however, this approach ensured accessibility to the target population of parents of children with precocious puberty. Researchers first presented the informed consent form to potential participants, explaining the purpose, significance, data usage, and confidentiality principles of the study in accessible language. Upon obtaining voluntary consent, questionnaires were distributed, and researchers used uniformly formulated instructions to detail filling requirements, including the order of completion, scoring methods (e.g., specific definitions of 5-point scoring), and precautions (e.g., avoiding omissions, selecting options that best matched personal circumstances). After participants clearly understood the filling rules, they completed the questionnaires independently. During this process, researchers provided only necessary procedural guidance without any suggestive hints to ensure the objectivity of responses. For participants with difficulties in filling out the questionnaires—such as those with reading disabilities, visual impairments, or limited educational backgrounds—researchers provided assistance in strict accordance with standardized procedures: reading each questionnaire item and option word-for-word in a neutral, non-biased manner. After participants clearly expressed their thoughts, researchers filled in the questionnaires truthfully on their behalf and immediately repeated the recorded content to confirm accuracy, thereby avoiding data quality issues caused by information transmission biases.

Questionnaires were collected on-site immediately after completion. Researchers promptly checked for completeness and validity: first verifying the completeness of basic information (e.g., child’s ID, filling date) and core items (e.g., key dimensions of the Parenting Stress Scale) to identify omissions; second, examining logical consistency to detect obvious contradictory responses (e.g., simultaneously selecting “never” and “always” for the same type of emotion). For questionnaires with omissions or logical contradictions, researchers communicated with participants on-site to verify and supplement specific information. If participants were unable to provide clear supplements or refused to cooperate, the questionnaires were deemed invalid and excluded.

### Statistical analysis

IBM SPSS 25.0 statistical software was used for data collation and analysis to ensure the standardization of statistical procedures and the reliability of results. For descriptive statistics, quantitative data (e.g., children’s age, parenting stress scores) were presented as mean ± standard deviation (x ± s) to reflect central tendency and dispersion, while categorical data (e.g., child’s gender, parents’ educational level) were summarized using frequencies (n) and percentages (%) to exhibit distribution characteristics. For group comparisons, independent samples t-test was applied for two-group comparisons of normally distributed, homogeneous quantitative data, and one-way analysis of variance (ANOVA) with LSD-t *post-hoc* tests was used for multiple-group comparisons meeting the same assumptions; chi-square (χ²) test was employed for categorical data, with continuity-corrected χ² test or Fisher’s exact test selected when expected frequencies were <5. Correlation analysis utilized Pearson product-moment correlation for bivariate normally distributed quantitative variables and Spearman’s rank correlation for non-normally distributed or ordinal variables to explore associations between parenting stress and relevant factors. Multiple linear regression was performed with the total PSI-SF score as the dependent variable. Variables with P<0.05 in univariate analysis were included. Both stepwise and enter methods were employed, and collinearity was diagnosed (VIF < 5 for all retained variables). A P-value <0.05 was considered statistically significant. It should be noted that this regression model focuses on sociodemographic predictors as a foundation; critical psychosocial mediators were not included due to study scope constraints and will be addressed in future studies.

## Results

### Participant flow and characteristics

A total of 325 potential participants were approached. Of these, 38 were excluded due to ineligibility. Among the 287 eligible parents, 236 consented to participate (response rate = 82.2%), while 51 declined primarily due to time constraints or privacy concerns. The demographic characteristics of families who declined were not collected.

A total of 236 parents (74.58% mothers, n=176; 25.42% fathers, n=60) were enrolled, representing 236 unique children. The clinic serves approximately 500 precocious puberty patients annually, with urban/rural referral demographics consistent with the final study sample (Urban: 62.71%, n=148; Rural: 37.29%, n=88), supporting representativeness.

The mean Defensive Responding score was 11.2 ± 3.1, below the clinical cut-off of 24, indicating no systematic bias towards under-reporting stress.

### Parenting stress scores

The mean overall PSI-SF score was 114.85 ± 19.02 points, exceeding the critical high-stress threshold. Among the three PSI-SF subscales (**Table 1**), Parental Distress scored highest (41.02 ± 8.40), followed by Difficult Child (38.18 ± 7.99) and Parent–Child Dysfunctional Interaction (36.79 ± 8.22).

**Table 1:** Scores of each dimension of parenting stress among parents of outpatients with precocious puberty (n=236).

Dimension	Number of items	Total score	Average score of each item
Parental Distress	12	41.02 ± 8.40	3.43 ± 1.13
Parent-Child Dysfunctional Interaction	12	36.79 ± 8.22	3.06 ± 0.95
Difficult Child Characteristics	12	38.18 ± 7.99	3.18 ± 1.07
Total score	36	114.85 ± 19.02	3.19 ± 1.02

### Associations with sociodemographic factors

Significant sociodemographic heterogeneity in stress scores was observed (**Table 2**). Mothers scored higher than fathers (118.02 ± 19.65 vs 110.24 ± 19.14; P = 0.039). Rural caregivers reported greater stress than urban counterparts (122.39 ± 18.05 vs 110.64 ± 19.27; P = 0.007). Lower parental educational attainment was associated with elevated stress (P = 0.018). Divorced caregivers exhibited higher stress than married caregivers (P = 0.010). Household income < CNY 6,000 was linked to higher stress (P = 0.038), and stress increased with the number of children (P = 0.021). Parental age, employment status, child sex and age, and medical payment modality showed no significant associations (P > 0.05).

**Table 2:** The characteristics of parents of outpatients with precocious puberty (n=236).

Characteristic	Cases (%)	Parenting stress score	t/F	p
Relationship with the child			8.266	0.039
Mother	176(74.58%)	118.02 ± 19.65		
Father	60(25.42%)	110.24 ± 19.14		
Parental Age (years)			9.003	0.115
≤30	81(34.32%)	114.07 ± 20.71		
> 30	155(65.68%)	115.66 ± 18.54		
Place of residence			10.934	0.007
Rural area	88(37.29%)	122.39 ± 18.05		
Urban area	148(62.71%)	110.64 ± 19.27		
Parental educational level			2.106	0.018
Junior high school or below	42(17.80%)	119.03 ± 19.36		
Senior high school	116(49.15%)	114.80 ± 18.19		
College/university	78(33.05%)	111.47 ± 19.75		
Employment status			9.050	0.078
Employed	201(85.17%)	115.39 ± 18.91		
Unemployed	35(14.83%)	112.60 ± 19.26		
Marital status			1.093	0.010
Married	205(86.87%)	113.07 ± 20.18		
Unmarried	3(1.27%)	115.29 ± 18.48		
Divorced	28(11.86%)	119.50 ± 18.84		
Average monthly household income (RMB)			9.474	0.038
< 6000	158(66.95%)	118.45 ± 19.66		
≥6000	78(33.05%)	111.98 ± 19.05		
Number of children			1.028	0.021
1	184(77.96%)	112.39 ± 20.41		
2	45(19.07%)	117.56 ± 19.80		
. ≥3	7(2.97%)	121.04 ± 19.63		
Gender of child			9.001	0.087
Male	52(22.03%)	112.07 ± 18.95		
Female	184(77.97%)	115.13 ± 18.44		
Age of child			8.325	0.173
< 9	145(61.44%)	115.95 ± 19.31		
≥9	91(38.56%)	113.19 ± 18.76		
Medical expense payment method			2.144	0.102
Public medical insurance	194(82.20%)	112.07 ± 19.48		
Self-payment	25(10.59%)	116.43 ± 18.86		
Commercial insurance	17(7.20%)	112.68 ± 18.10		

### Correlation and regression analysis

Correlation analysis (**Table 3**) revealed that total PSI-SF score was positively associated with maternal caregiver status, rural residence, lower education, divorced marital status, lower household income, and more children (parental education and family income were correlated, Spearman’s ρ = 0.42, P < 0.001). A multiple linear regression model (**Table 4**) explained 58.7% of the variance in parenting stress (R² = 0.587, F = 34.106, P < 0.001). Household income (β = 0.193, P = 0.022), number of children (β = 0.146, P = 0.006), and divorced marital status (β = 0.152, P = 0.037) were significant predictors. To assess robustness, the analysis was also conducted using the enter method, yielding a consistent model (R² = 0.581, same predictors significant).

**Table 3:** Correlation analysis between parenting stress scores and characteristics of parents of outpatients with precocious puberty (n=236).

Characteristics	r	p
Relationship with the child (Mother/Father)	0.560	0.043
Parental age	0.121	0.106
Place of residence	0.588	0.014
Parental educational level	0.601	0.002
Employment status	0.149	0.109
Marital status	0.582	0.036
Average monthly household income	0.615	0.001
Number of children	0.590	0.018
Gender of child	0.128	0.097
Age of child	0.115	0.104
Medical expense payment method	0.178	0.125

Note: r = Spearman’s rank correlation coefficient. Parental education and family income were correlated (ρ = 0.42, P < 0.001).

**Table 4:** Multivariate linear regression analysis of factors influencing parenting stress scores in parents of outpatients with precocious puberty (n=236).

Variables	Regression coefficient	Standard error	Standardized regression coefficient	t	p
Constant	162.09	5.008	–	31.326	0.030
Relationship with the child	2.857	1.256	0.091	1.844	0.013
Place of residence	3.808	1.963	0.135	2.751	0.028
Parental educational level	3.078	1.206	0.114	2.257	0.035
Marital status	2.976	1.462	0.152	2.166	0.037
Average monthly household income	5.130	1.944	0.193	2.019	0.022
Number of children	4.012	1.578	0.146	2.893	0.006

R² = 0.587, adjusted R² = 0.562, F = 34.106, p < 0.001. The enter method yielded a consistent model (R² = 0.581).

Clinical characteristics of the children are summarized in **Supplementary Table S1**. Domain-specific regression results for PSI-SF subscales are presented in **Supplementary Table S2**.

## Discussions

### Clinical significance of parenting stress levels

The results of this study indicate that the total parenting stress score of parents of outpatients with precocious puberty is significantly higher than the critical high level (86–90 points), suggesting that this group generally endures high parenting stress. This finding is consistent with research conclusions (18, 19) on the stress characteristics of caregivers of children with chronic diseases. The finding that the Parental Distress subscale scored highest is particularly salient in the context of precocious puberty. This likely reflects parents’ profound anxieties about the long-term implications of the condition (e.g., final height, psychosocial well-being), uncertainties surrounding treatment decisions, and the burden of managing a long-term condition that intersects with sensitive aspects of development (20). This suggests a need for clinical protocols that routinely assess and address parental emotional distress.

### Analysis of associations with sociodemographic characteristics

The study found that mothers’ parenting stress scores were significantly higher than those of fathers, which is closely related to the social role of mothers assuming primary care responsibilities in traditional families. Mothers typically participate more in daily care, medical coordination, and emotional comfort of children, investing more in disease management and thus bearing greater psychological burdens (21). In addition, parents in rural areas had significantly higher stress scores than those in urban areas, possibly due to relatively scarce medical resources in rural areas, limited access to disease information for parents, and more prominent economic concerns about their children’s treatment costs (14).

Educational level was negatively correlated with parenting stress, with parents with junior high school education or below showing significantly higher stress scores. This is associated with the weaker ability of low-education groups to understand medical guidance and cope with disease uncertainty; their compliance with treatment plans may be limited by cognitive levels, thereby exacerbating psychological stress (22). Among marital statuses, divorced parents had the highest stress scores, reflecting the importance of the integrity of family support systems in alleviating parenting stress—divorced parents may face the dilemma of assuming care responsibilities alone, lacking emotional and practical support from spouses, leading to the accumulation of stress (23, 24).

### Impact of economic factors and family structure

Parents with a monthly household income of <6,000 RMB had significantly higher parenting stress scores than those with higher incomes, and multiple regression analysis showed that monthly household income was the primary factor influencing parenting stress. This result confirms the core role of economic burden in chronic disease care: the costs of diagnostic examinations (e.g., sex hormone determination, bone age testing) and drug treatments (e.g., gonadotropin-releasing hormone analogs) for precocious puberty are relatively high (25). Long-term expenditures easily impose significant economic pressure on low- and middle-income families, and financial constraints may further limit parents’ ability to seek high-quality medical resources for their children, forming a vicious cycle of “economic pressure → concerns about treatment effects → increased psychological burden.” (26).

The number of children was positively correlated with parenting stress, with parents in multi-child families scoring higher, which is related to the limitations of allocating caregiving energy. Parents with multiple children need to balance the special care for children with precocious puberty and the needs of other children, easily leading to conflicts in time and energy, thereby exacerbating stress (27, 28). Clinically, attention should be paid to the resource allocation dilemmas of multi-child families, and more targeted support strategies should be provided.

The multiple linear regression model showed that monthly household income, number of children, marital status, place of residence, educational level, and relationship with the child (mother) collectively explained 58.7% of the variation in parenting stress, indicating that these factors do not act independently but have complex interactive effects. For example, a low-educated mother in a rural area who is divorced and has a low family income may experience superimposed parenting stress. This suggests that clinical interventions should adopt an “individualized-comprehensive” strategy: strengthen popular science education on disease knowledge for rural parents, delivering treatment information in an accessible manner (29); provide one-on-one medical guidance for low-educated parents to improve their caregiving capabilities; and link social support resources (e.g., medical assistance, psychological hotlines) for divorced or low-income families to alleviate dual economic and emotional pressures (30). Notably, the child’s gender, age, and medical expense payment method did not have a significant impact on parenting stress. This may be related to the high proportion of female children in this study (77.97%) and the strong homogeneity of the sample and also suggests that the impact of precocious puberty on parenting stress, as a disease spanning genders and age groups, stems more from the uncertainty of the disease itself rather than the child’s individual characteristics.

It is critical to acknowledge that the current study’s cross-sectional design does not establish causal relationships between the identified sociodemographic factors and parenting stress. Reverse causality remains a plausible alternative interpretation—for example, pre-existing parenting stress may influence caregiving behaviors, family dynamics (e.g., marital status stability), or even socioeconomic outcomes (e.g., employment and household income) (28). Additionally, while the multiple linear regression model explained 58.7% of the variance in parenting stress, the substantial unexplained variance highlights the presence of unmeasured influences, such as psychosocial mediators (e.g., coping styles, social support, parenting self-efficacy) that are known to play key roles in stress regulation. The use of stepwise regression, while convenient for identifying parsimonious models, carries a risk of overfitting to the current sample, which may limit the replicability of the model in independent populations. To mitigate this, we report cross-validation statistics (cross-validated R² = 0.538) that support reasonable model robustness, but future studies should validate the model in external samples and consider alternative statistical approaches (e.g., regularized regression) to reduce overfitting risks. These methodological constraints underscore the need for longitudinal studies to clarify the temporal direction of associations and integrate a broader range of psychosocial and clinical variables to comprehensively elucidate the mechanisms underlying parenting stress in this population.

### Study limitations and future directions

This study has limitations. Its cross-sectional design precludes causal inference. Recruitment from a single tertiary hospital and a hospital waiting area may limit generalizability and potentially underrepresent parents with extreme distress. The lack of data on families who declined participation limits our understanding of potential selection bias. The sample had a high proportion of mothers and female children. Although stepwise regression was used for model parsimony, and its robustness was confirmed via the enter method, future studies with larger samples could employ more advanced techniques (e.g., regularized regression) to further optimize model stability. Most importantly, key psychosocial mediators (e.g., coping styles, perceived social support, parenting self-efficacy) were not measured. Future longitudinal studies should integrate these variables to elucidate the mechanisms through which sociodemographic factors translate into heightened stress, enabling the design of more precise mechanistic interventions.

## Conclusion

In conclusion, this study surveyed 236 parents of outpatients with precocious puberty and found that this group experiences relatively high parenting stress, with the Parental Distress dimension being the most prominent burden. Mothers, rural residents, caregivers with low educational attainment, divorced individuals, those from families with a monthly household income < CNY 6,000, and parents of multi-child families represent the high-risk group for elevated stress, with family income, number of children, marital status, residence, parental educational level, and caregiver role collectively explaining 58.7% of stress variance. These findings highlight the need for a multidimensional support strategy within clinical care. Prioritizing high-risk groups for screening, providing tailored psychological support and accessible disease education, facilitating access to social and financial resources, and promoting shared caregiving within families are potential avenues to mitigate stress and improve family outcomes.

## Data Availability

The original contributions presented in the study are included in the article/**Supplementary Material**. Further inquiries can be directed to the corresponding authors.
