# Supplementary material for: Parenting stress among parents of outpatients with precocious puberty: a cross-sectional survey
Source: Front Psychiatry. 2026 Feb 24;17:1688507. doi: 10.3389/fpsyt.2026.1688507 (PMC12971896; doi:10.3389/fpsyt.2026.1688507)
Supplement: Supplementary file 1 [file SupplementaryFile1.docx]

Supplementary Table 1 Clinical and Demographic Characteristics of Participating Children (n = 236)

| Characteristic | Category/Detail | n | % | Mean ± SD |
| --- | --- | --- | --- | --- |
| Gender | Female | 184 | 77.97 | — |
|  | Male | 52 | 22.03 | — |
| Age (years) | Overall | — | — | 7.8 ± 1.2 |
|  | < 7 years | 98 | 41.53 | — |
|  | 7–8 years (girls) / 7–9 years (boys) | 112 | 47.46 | — |
|  | > 8 years (girls) / > 9 years (boys) | 26 | 11.02 | — |
| Pubertal Stage (Tanner) | II | 156 | 66.10 | — |
|  | III | 65 | 27.54 | — |
|  | IV | 15 | 6.36 | — |
| Treatment Status | Observation only | 84 | 35.59 | — |
|  | Gonadotropin-Releasing Hormone Analogs (GnRH-a) | 152 | 64.41 | — |
| Disease Duration (months) | Overall | — | — | 8.3 ± 3.1 |
|  | ≤ 6 months | 103 | 43.64 | — |
|  | 7–12 months | 91 | 38.56 | — |
|  | > 12 months | 42 | 17.80 | — |
| Type of Precocious Puberty | Central Precocious Puberty (CPP) | 214 | 90.68 | — |
|  | Peripheral Precocious Puberty (PPP) | 22 | 9.32 | — |

Supplementary Table 2. Domain-Specific Correlations and Multiple Linear Regression Results for PSI-SF Subscales (n = 236)

| Predictor Variable | Parenting Distress | Difficult Child | Parent–Child Dysfunctional Interaction |
| --- | --- | --- | --- |
| Maternal caregiver status (Mother=1/Father=0) | r = 0.512 (P=0.038) β = 0.140 (P=0.047) | r = 0.489 (P=0.045) β = 0.118 (P=0.061) | r = 0.536 (P=0.032) β = 0.147 (P=0.040) |
| Rural residence (Rural=1/Urban=0) | r = 0.574 (P=0.011) β = 0.179 (P=0.020) | r = 0.541 (P=0.017) β = 0.129 (P=0.058) | r = 0.528 (P=0.021) β = 0.136 (P=0.053) |
| Low parental education (≤High school=1/College=0) | r = 0.586 (P=0.008) β = 0.169 (P=0.029) | r = 0.553 (P=0.015) β = 0.143 (P=0.051) | r = 0.603 (P=0.006) β = 0.187 (P=0.020) |
| Divorced marital status (Divorced=1/Other=0) | r = 0.558 (P=0.023) β = 0.156 (P=0.038) | r = 0.591 (P=0.010) β = 0.172 (P=0.030) | r = 0.532 (P=0.026) β = 0.134 (P=0.064) |
| Low household income (<CNY 6,000=1/≥CNY 6,000=0) | r = 0.621 (P=0.003) β = 0.206 (P=0.010) | r = 0.587 (P=0.009) β = 0.152 (P=0.042) | r = 0.594 (P=0.007) β = 0.164 (P=0.036) |
| Number of children (≥2) (≥2=1/1=0) | r = 0.569 (P=0.019) β = 0.145 (P=0.043) | r = 0.605 (P=0.007) β = 0.158 (P=0.020) | r = 0.547 (P=0.016) β = 0.123 (P=0.072) |
| Regression Model Fit | R² = 0.542, F = 29.741, P < 0.001 | R² = 0.518, F = 27.358, P < 0.001 | R² = 0.535, F = 28.914, P < 0.001 |

Note:

- r = Spearman’s rank correlation coefficient. β = Standardized regression coefficient from the multiple linear regression model for each subscale.
- All regression models were constructed using the enter method and adjusted for parental age and employment status.
- Only variables with P < 0.05 in the preliminary univariate correlation analysis for the respective subscale were included as predictors in its corresponding regression model.
